# Using Technology to Advance Cancer Survivorship Programs

**Published:** 2014-09-01

**Authors:** Mark Fosdal

**Affiliations:** Dr. Fosdal is currently a Medical Science Liaison for Pharmacyclics Pharmaceuticals; views expressed in this article are his alone and not those of Pharmacyclics. He was formerly a physician assistant at the Fred Hutchinson Cancer Research Center, Seattle, Washington.

Over 12 million people in the United States are surviving after being diagnosed with cancer; this number is expected to rise as baby boomers age and emerging targeted therapies create less toxicity (Ganz, 2009). Yet in a 2012 survey, only 55% of primary care providers (PCPs) were comfortable being the sole provider for cancer patients 2 years after the completion of their therapy (Salz, Oeffinger, McCabe, Layne, & Bach, 2012). Another survey that year stated that 84% of PCPs were unsure about the type, frequency, and duration of surveillance testing required for breast and colon cancer patients, and almost 50% were not comfortable monitoring for late complications of cancer and its treatment (Salz et al., 2012).

The cancer survivor’s transition from being cared for by an oncologist to a PCP was formally assessed by the Institute of Medicine (IOM) in its 2005 report "From Cancer Patient to Cancer Survivor: Lost in Transition" (IOM, 2005). The report’s recommendations for improvement of the process emphasized a smoother transition between the oncologist and referring physician, with more patient empowerment and involvement.

Some of the recommendations for the medical community included (1) implementing a comprehensive survivorship care plan summarizing cancer treatments and details for follow-up, (2) creating and disseminating evidence-based guidelines for screening and managing late complications, (3) seeking reimbursement from third-party payers for survivorship services, and (4) cooperating with both public and private agencies in the research and awareness of survivorship programs (Ganz, 2009). Since the IOM recommendations were published, the Commission on Cancer of the American College of Surgeons has put forth new standards for accrediting hospital cancer programs, where improvement in ensuring patients a smooth transition back to their referring physician is needed (Voelker, 2011).

Most oncologists agree (at least in theory) that creating a care plan is important for meeting the IOM recommendations, but they often struggle to choose a concise, workable format and to find the time, personnel, and resources to complete an individual plan for each patient (Salz et al., 2012). Comprehensive cancer centers often receive funding to develop survivorship programs from patient advocacy groups such as the Livestrong Foundation and the National Cancer Institute (NCI) in the hope of paving the way for community practices. There is a consensus that this will require a variety of models, as each practice has limited resources to address its unique patient populations (Ganz, 2009).

The term "cancer survivorship" can encompass many different phases of treatment, starting from the time of diagnosis through hospice care. An understanding of the patient population being served is fundamental when discussing the initiation of survivorship care. Resources for understanding general guidelines and different types of survivorship programs can be found in the Table.

**Table 1:**
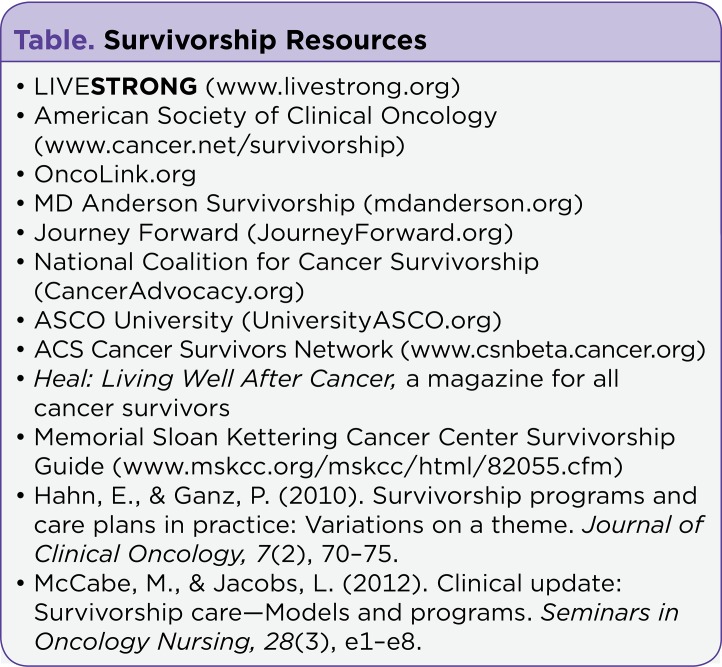
Table. Survivorship Resources

Patients receiving treatment for cancer represent less than 1% of the commercially insured population but account for approximately 10% to 12% of health care expenditures (Sprandio, 2010). The extreme cost of oncology care was in part created by inefficiencies related to communication and coordination of care provided by multiple providers in different health care systems. With the passing of the Affordable Care Act, the clinician’s role continues to expand, yet reimbursements for these changes lag behind.

The use of technology is one way of working toward making the delivery of care more cost-effective. Although more complex systems have incorporated either electronic medical records (EMRs) or personalized care plans to facilitate care for cancer survivors, educating survivors through Web-based tools is becoming standard. But how can emerging and established technologies be used in survivorship programs to incorporate efficiency with patient satisfaction yet remain economically feasible?

To better understand how to enhance and improve survivorship programs through technology, a review of the literature was done. This process attempted to illuminate (1) how patients and clinicians currently use the Internet, (2) how specific software is used to improve oncology care, and (3) what strategies survivorship programs have used to incorporate the Internet to improve care. This understanding could lay the foundation for future research and implementation of newer models for survivorship programs.

During the literature review, the analysis encompassed a narrower definition of patients: those actively being treated for cancer, including those being transitioned back to the referring physician. This population required facilitation of care among the oncologist, the referring physician, and the patient, where the Internet or other forms of technology had shown an impact in patient outcomes. The focus of this literature review did not consider facilitating treatment while the patient was in hospice or receiving palliative care.

## Review of the Literature

**Internet Use by Cancer Survivors and Clinicians**

Internet use among patients with cancer has increased during the past decade. Dissatisfaction with information and communication during clinical encounters with health-care professionals, among other factors, has propelled cancer survivors to search for information on the Internet. This dissatisfaction may have resulted from a lack of face-to-face time with their providers or the fact that the information provided by their clinicians was outdated (Dolce, 2011). In one survey, the Internet was demonstrated to be the most used source of information for breast cancer survivors, whereas the use of other sources (books, videos) tended to decline from the time of diagnosis (Hill-Kayser, Vachani, Hampshire, & Metz, 2011).

Several oncology practices have implemented technology into their outreach programs, with varying degrees of success. Educational materials can be found on clinic websites, including transcripts of oncology seminars, YouTube interviews, podcasts, and links to accredited oncology resources (Kaplan, 2012). Patient advocacy groups such as Susan G. Komen for the Cure or the Livestrong Foundation have also created Internet materials, including pdfs, podcasts, and webinars, which can assist in patient education and may empower greater participation during conversations with health-care providers (Livestrong Foundation, 2012; Susan G. Komen for the Cure, 2012).

Other clinics have used social media to facilitate conversations between patients. The Mayo Clinic, for example, created a Facebook page to assist patients newly diagnosed with Barrett’s high-grade dysplasia and esophageal cancer as they anticipated surgical and postoperative challenges. Initially, 65 patients were enrolled; the page provided mutual support for managing treatment effects or nutritional challenges after surgery. The physicians did not give medical advice through the website but were able to update patients with newer technologies and newsletters while using information from patient posts to facilitate treatment strategies during clinic visits. After the initial success of this support group, the oncology staff enrolled more patients, who participated in sharing best practices and insights about coping with treatments and outcomes (Kaplan, 2012).

**Oncology Practices Using Software to Improve Care**

One of the more comprehensive implementations of technology discussed in recent literature involved a group of physicians who integrated an oncology-based software system within their private practice (Sprandio, 2010). This nine-physician practice worked with three separate health care systems using customized oncology software. The practice was able to facilitate patient care while mining pertinent information that reported trends in patient symptoms and treatment.

The implementation of this software allowed a more personalized approach, and clinicians were able to identify and treat patients at higher risk for complications. A nurse phone triage system, for example, was created to identify, track, and manage symptoms such as dehydration, diarrhea, and insomnia. Identifying such symptoms allowed nurses and patient navigators to facilitate referrals with community services more proactively, thus avoiding patient morbidity and clinic resource utilization. Another essential feature this software integration was physician documentation. Consulting physicians had portal access to facilitate prompt communication, with up-to-date information about treatments and other comorbid issues (Sprandio, 2010).

After the practice used this software for 5 years, it had lowered patient emergency room (ER) visits by an estimated 68%, reduced hospital admissions treated with chemotherapy by 51%, and reduced length of stay for admitted patients by 21%. The practice also had seen a 22% reduction in outpatient visits/year in the hematology/oncology population and a 12% reduction in outpatient visits/year in the chemotherapy subpopulation (Sprandio, 2012).

One component critical to this model, as noted by the author, was adequate third-party reimbursement for what was considered more efficient care. In the present fee-for-service reimbursement system, fewer ER visits or less chemotherapy administered means less revenue for the hospital and oncology staff. Without a change in the reimbursement structure, this type of innovative care delivery system would not be financially viable. The author mentioned ongoing discussions with insurance companies regarding hybrid concepts that would incorporate bundle reimbursement plans to make this type of care financially feasible (Sprandio, 2010).

However, other experiences of incorporating newer technologies within oncology private practices have not been as successful (Piana, 2012). For instance, a private oncology practice with 4 physicians covering a community of 120,000 people implemented an EMR in their clinic. After a difficult transition of integrating software and an EMR system over a 3-year period, the practice hired technicians to interface with other systems; each software interface ranged from $20,000 to $40,000. The providers in this clinic described their system as "expensive, clunky, and inefficient," as it had taken away time available to spend with patients (Piana, 2012). Like many other private oncology practices, the financial strain caused this physician group to merge with a local hospital; the physicians’ time became more constrained, as they worked with two separate EMR systems to follow patients’ progress and document their notes. As technology continues to be integrated with patient care, collaboration with qualified technical support staff can mitigate some of the unexpected barriers, as successful integration has also been published (Syrjala et al., 2011).

**Internet Use With Survivorship Programs**

Cancer survivorship programs have incorporated treatment models using Internet- or software-based technology to facilitate a better quality of life for patients. After the IOM recommended individualized care plans for cancer survivors, the University of Pennsylvania launched a Web-based tool using survivorship care plans to provide comprehensive individualized plans for those with breast cancer (Hill-Kayser et al., 2011). During this trial, almost 4,400 care plans were generated, with a mean time of 3 years after initial diagnosis.

Similar to other studies, this trial showed a higher trend in users being Caucasian, younger, and more educated than other cohorts of breast cancer patients. Only 12% of these women had previously received survivorship information, and 17% of this same cohort could identify an established survivorship program (Hill-Kayser et al., 2011). There was heterogeneousness in survivorship expectations among breast cancer survivors. Almost 75% of the patients surveyed thought that either enough or not enough information was presented on this Internet-based care plan, the rest of the responders thought too much information was given. The authors stressed the importance of developing a variety of models to provide support and education, with the flexibility of adapting to the specific needs of any patient population (Hill-Kayser et al., 2011).

One academic center assessed patients’ ability to enroll in and use an Internet educational program after receiving a bone marrow transplant (BMT; Syrjala et al., 2011). Currently, most patients are no longer followed by their oncologists 2 to 5 years after transplant. Yet primary care providers have verbalized a lack of understanding of this complex setting, as this vulnerable patient population has a mortality rate four- to nine-fold higher than the non-BMT population (Syrjala et al., 2011).

This academic center formed a cross-functional team, which took 2 years to create and program an Internet site, including online registration, consent, assessment, and study implementation (Syrjala et al., 2011). In this study, 775 post-BMT patients from one institution were followed for the purpose of providing Internet-based support. Interventions focused on reducing comorbidities (cardiovascular or bone complications), restoring energy, and renewing outlook (managing psychosocial health) while providing resources for specific questions.

The results focused on the technical aspects of enrolling and maintaining an interactive website for those on study. Almost 60% of the participants required one phone conversation for technical questions about the online process, whereas almost 30% required multiple phone conversations. Once patients had mastered the technical aspects of the site, there was improved compliance in answering the questions and using available resources. The authors expect to broaden this interventional tool for patients from different cancer centers who need ongoing assessment after BMT, with further tailoring to include video and social-networking features (Syrjala et al., 2011).

## Discussion

Survivorship programs have a unique opportunity to incorporate the Internet and other software technologies, as this new area of managing cancer patients is in its infancy, with no concrete "brick-and-mortar" policies in place. Practitioners can learn from common themes, as results from these initial programs have been published (Chubak et al., 2012).

Because clinical trials using Internet-based intervention are relatively new, internal review boards must establish standard practices proactively to ensure efficient implementation of future similar clinical trials (Syrjala et al., 2011). This should ensure that patient interests are prioritized while a better understanding of the dynamics involved with technology and patient care emerges. As more is learned about Internet participation through trials, principal investigators can integrate updated standards, saving time and money, as newer strategies using the Internet or other software-delivery systems are tested.

Building Internet-based programs (compared with institution-based ones) using evidence-based medicine will be critical as we move forward in pioneering new ways of monitoring, educating, and intervening on the behalf of patients (Snyder et al., 2011). As mentioned previously, Syrjala and coworkers (2011) worked with a multidisciplinary team for 2 years to develop an Internet-based evaluation and intervention program for patients who had undergone BMT. Their plan of expanding this Web-based program to patients at other institutions is one example of applying efforts beyond a single site to include a regional or national pool of patients. This Internet-based system has the potential to allow physicians to use the same database without having to communicate with different EMRs. Another benefit of incorporating an Internet site with a national patient population would be the increased data-mining capabilities, thus allowing "real-time" feedback for research regarding patient outcomes, which could advance standard practices.

**Transitioning Back to Primary Care**

The model of using technology to transition patients from their oncologist back to their referring physician could be a prototype for managing other comorbidities such as cardiac disease or diabetes (Sprandio, 2012). Implementing oncology-based software with clinic visits is another way to identify and coordinate care as the patient transitions back to the referring physician.

Sprandio’s pilot study using oncology-specific software has allowed his clinic to improve its efficiency in caring for its patient population, as evidenced by decreased ER visits and shortened inpatient stays (Sprandio, 2010). By using such software, clinicians may more accurately and efficiently summarize the care provided in a personalized plan for both patients and referring physicians.

Sprandio’s aforementioned dialog with insurance companies toward replacing fee-for-service reimbursement with a form of bundled payments is a conversation being repeated in multiple settings, as our reimbursement system is undergoing changes under the Affordable Care Act (Kulkarni, 2012). Once there is a financial incentive for insurance companies to reimburse for the use and coordination of care using technology, software companies may be more active in identifying effective systems that would be both effective and affordable for community oncologists.

**Technology Comfort Levels**

Helping patients use Internet-based programs will continue to be a challenge, as there has been a wide range of patient participation (12%–54%) in Web-based care systems (Syrjala et al., 2011). Although the Internet is a primary source of information for many adults, cancer survivors tend to access the Internet less often than those without cancer; this may be due to fatigue or other symptoms associated with their malignancy or treatments. Patients should be encouraged to make use of available technology, which may allow proactive control of symptoms arising from treatment or underlying malignancies.

Clinicians should also be mindful of some patients’ limited capabilities in using technology or the Internet. More efficient care with effective interventions may, in part, depend on features of the Internet site or software programs. It may be necessary to provide technology support for patients who are not "technologically savvy" to aid in navigating any Internet-based program that requires participation, including accessing password-protected systems (Syrjala et al., 2011). Varied technology-delivery methods (computer, smartphone, tablet, etc.) and user-friendly website designs are other important features that would assist in patient enrollment and continued use (Salz et al., 2012).

Another disparity observed among cancer survivors using the Internet is that non-Caucasian ethnic groups may be less likely to use or have access to the Internet compared with other groups, perhaps as a result of a lower level of education, living in a rural area, or having poorer physical health (Salz et al., 2012). Efforts are being made to encourage a variety of ethnic groups to make use of such technology; for instance, templates have been created in Spanish for user-friendly systems such as the iPhone and some laptops (Hill-Kayser et al., 2011). During any transition toward an Internet-based system, it is important that survivorship care plans be provided in paper-based copies.

**The Impending Shortage of Oncologists**

The use of technology in caring for oncology patients may be hastened by the impending shortage of oncologists. Studies have shown that even 3 years after a diagnosis of cancer, 50% of patients are still being followed by their oncologist, 10% by their primary care physician, and approximately 40% by both (Hill-Kayser et al., 2011). By 2020, there may be an anticipated 48% increase in cancer incidence and an 81% increase in people living with or surviving cancer. Meanwhile, the supply of oncologists is expected to grow only by 14%, leaving a shortage of between 2,500 and 4,000 oncologists by the next decade (Levit, Smith, Benz, & Ferrell, 2010).

Given the impending shortage of oncologists, an option for efficient delivery of care in transitioning the patient back to the primary care physician is to maximize the educational level of the clinical staff when assigning routine tasks (Lin & Donehower, 2010). The use of clinicians for nonclinician roles in survivorship programs should be reassessed, as oncology practices strive to stay financially viable.

For example, one survey of nurses reported that 16% provided employment assistance or legal issues while setting aside other nursing responsibilities (such as creating a personalized care plan; Irwin, Klemp, Glennon, & Frazier, 2011). Having more specialized personnel, such as social workers or patient navigators, is one way to delegate responsibility properly. This approach would allow nurses time for more appropriate tasks such as collecting pertinent material to formulate a personalized care plan prior to the patient visit.

Another option for optimizing care in survivorship programs with the shortage of oncologists is expanding the role of advanced practitioners (APs), including nurse practitioners and physician assistants. As survivorship programs are developed, more centers appear to incorporate APs in the patient care role, as physicians continue to see patients in the active treatment setting while remaining available for consultation (Oeffinger & McCabe, 2006; McCabe & Jacobs, 2012).

The American Society of Clinical Oncology (ASCO) commissioned a study showing the emerging importance of incorporating APs in the oncology setting (Polansky, Ross, & Coniglio, 2010). Productivity was shown to be highest when using physician assistants or nurse practitioners in advanced activities. Essential tasks for APs include discussing late effects of chemotherapy (including risk for secondary cancers), assessing psychosocial needs based on the IOM recommendations, and coordinating these results and recommendations with primary care physicians. By delegating care plan development and coordinating patient care to the appropriate staff, oncologists can use their time for management that requires a certain level of expertise.

**Staff Training and Support**

Because survivorship programs require a team approach, adequate education of both treatment-based complications and the proper use of any Internet site or software-based programs is needed. In a study from the Oncology Nursing Society, only 27% of 399 nurses surveyed worked in settings with a formal survivorship program (Irwin et al., 2011). The greatest barriers for the nurses were lack of time and funding. For nurses with less than 5 years of oncology experience, the majority stated they lacked sufficient knowledge in providing the needed care to this population in transition. Acquainting clinical staff who may not be familiar with survivorship literature to pertinent resources may aid in retention of staff members and improvement in the quality of care provided.

One recommendation to provide educational programs for nurses new to adult survivorship programs is to learn from established pediatric programs, where there is a larger experience base in monitoring long-term complications. Only 33% of nurses working exclusively in adult settings said that patients were provided a written summary and follow-up care plan, vs. 70% of those in pediatric settings (Irwin et al., 2011). These pediatric programs identified the importance of defining the roles of primary and specialty physicians, attending to insurance issues, and obtaining a treatment summary and recommendations from primary oncologists.

## Conclusion

The creation of survivorship clinics could not come at a better time, as the overall incidence of cancer is estimated to increase by 40% among women and 55% among men by 2020 (Lichtenfeld, 2009). As academic and community clinics develop survivorship programs that are both financially feasible and beneficial to patients, studies have shown that online surveys can be less expensive; reach larger and more widely dispersed study populations; and increase the accuracy, completeness, and consistency of data collection compared with paper versions (Ahern, 2005).

Creating Internet-based survivorship programs to facilitate the transition of care back to referring physicians is one facet of care that is still in its infancy. Until data elements conform to common standards, are communicated easily from oncologists to primary care providers, and are placed in other providers’ EMRs, the goal of fully integrating technology and care of cancer patients will not be achieved. Clinical trials currently under way may reach a broader group of patients, as more of them become comfortable using technology to complement their present care during this transition period. Moving away from institution-based technology systems toward Internet-based systems will also allow more patients to participate, thus increasing data banks where stored information is retrieved; emerging standards of care can be implemented with more efficient clinical pathways for cancer survivors transitioning back to their referring physicians. 
